# Small RNA Profiles of Serum Exosomes Derived From Individuals With Latent and Active Tuberculosis

**DOI:** 10.3389/fmicb.2019.01174

**Published:** 2019-05-28

**Authors:** Lingna Lyu, Xiuli Zhang, Cuidan Li, Tingting Yang, Jinghui Wang, Liping Pan, Hongyan Jia, Zihui Li, Qi Sun, Liya Yue, Fei Chen, Zongde Zhang

**Affiliations:** ^1^Beijing Key Laboratory for Drug Resistant Tuberculosis Research, Beijing Tuberculosis and Thoracic Tumor Research Institute, Beijing Chest Hospital, Capital Medical University, Beijing, China; ^2^CAS Key Laboratory of Genome Science and Information, Beijing Institute of Genomics, Chinese Academy of Sciences, Beijing, China; ^3^University of Chinese Academy of Sciences, Beijing, China

**Keywords:** exosome, miRNA, tuberculosis, RNA sequencing, small RNA, latent TB infection

## Abstract

Tuberculosis (TB) has been the leading lethal infectious disease worldwide since 2014, and about one third of the world’s population has a latent TB infection (LTBI). This is largely attributed to the difficulties in diagnosis and treatment of TB and LTBI patients. Exosomes offer a new perspective on investigation of the process of TB infection. In this study, we performed small RNA sequencing to explore small RNA profiles of serum exosomes derived from LTBI and TB patients and healthy controls (HC). Our results revealed distinct miRNA profile of the exosomes in the three groups. We screened 250 differentially expressed miRNAs including 130 specifically expressed miRNAs. Some miRNAs were further validated to be specifically expressed in LTBI (hsa-let-7e-5p, hsa-let-7d-5p, hsa-miR-450a-5p, and hsa-miR-140-5p) and TB samples (hsa-miR-1246, hsa-miR-2110, hsa-miR-370-3P, hsa-miR-28-3p, and hsa-miR-193b-5p). Additionally, we demonstrated four expression panels in LTBI and TB groups, and six expression patterns among the three groups. These specifically expressed miRNAs and differentially expressed miRNAs in different panels and patterns provide potential biomarkers for detection/diagnosis of latent and active TB using exosomal miRNAs. Additionally, we also discovered plenty of small RNAs derived from genomic repetitive sequences, which might play roles in host immune responses along with *Mtb* infection progresses. Overall, our findings provide important reference and an improved understanding about miRNAs and repetitive region-derived small RNAs in exosomes during the *Mtb* infectious process, and facilitate the development of potential molecular targets for detection/diagnosis of latent and active tuberculosis.

## Introduction

According to WHO Reports, tuberculosis (TB) has been the leading lethal infectious disease worldwide since 2014 (World Health Organization [WHO], 2018). Moreover, 1.7 billion (23%) of the world’s population have latent TB infection (LTBI), 5–10% of which will develop active TB disease during their lifetime (World Health Organization [WHO], 2018). TB is mainly caused by an intracellular bacterial pathogen *Mycobacterium tuberculosis* (*Mtb*). After *Mtb* infection, *Mtb* bacteria are phagocytosed by human macrophages: some of the intracellular *Mtb* bacteria are destroyed by macrophages; some of them are latent in cells by immune escape mechanism; some of them continue dividing and proliferating (Zhu et al., 2016; Jia et al., 2017; Huang et al., 2018; Yang et al., 2018). The “struggle” between *Mtb* strains and host macrophages results in three outcomes: health, LTBI and TB infection. Although previous studies have revealed some features of the “struggle" (Tufarielloa et al., 2003), more analysis is still needed to comprehensively clarify the process of TB infection.

Exosomes offer a new perspective on investigation of the process of TB infection. Exosomes are a particular pool of extracellular small membrane vesicles (∼100 nm) that are secreted into many body fluids (e.g., blood, urine, and pleural fluid) by eukaryotic cells. Previous research has reported that exosomes contain multiple biological molecules (e.g., proteins, lipids, and nucleic acids), and are involved in many physiological and pathological processes (e.g., cancer, diabetes, AIDS, and TB) by intercellular material transportation and signal transduction (Schorey and Bhatnagar, 2008; Alipoor et al., 2016).

Some research concerning TB revealed that both *Mtb* and host derived cargoes could be delivered by exosomes (Kruh-Garcia et al., 2014). They focused on the functional analysis of exosomal miRNAs and development of potential non-invasive biomarkers for TB diagnosis using exosomal miRNAs (Bhatnagar and Schorey, 2007; Bhatnagar et al., 2007; Cheng and Schorey, 2007; Giri and Schorey, 2008; Giri et al., 2010; Singh et al., 2015). However, it is still necessary to comprehensively analyze the small RNA (including miRNA) of exosomes in human clinical specimens of TB and LTBI patients.

In this study, we analyzed small RNA (especially miRNA) profiles of serum exosomes derived from LTBI and TB patients and compared them to healthy controls (HC). We revealed distinct miRNA profiles of the exosomes from LTBI, TB, and HC groups, indicating the selective packaging of miRNA cargoes into exosomes under different stages of *Mtb* infection. In particular, we identified some specifically expressed miRNAs, and differentially expressed miRNAs belonging to different panels and patterns in the three groups, which provided potential biomarkers for detection/diagnosis of latent and active TB using exosomal miRNAs. Additionally, we also discovered plenty of small RNAs derived from genomic repetitive sequences (e.g., SINEs: short interspersed nuclear elements, LINEs: long interspersed nuclear elements, and LTR: long terminal repeat), which might play roles in host immune responses along with *Mtb* infecting progresses. Overall, our findings provide important reference and improved understanding about miRNAs and repetitive region-derived small RNAs in exosome during *Mtb* infectious process, and facilitate the development of potential molecular targets for detection/diagnosis of latent and active tuberculosis.

## Materials and Methods

### Patient Information and Sample Preparation

One hundred and eighty people were screened in this study and belong to 3 groups: (1) a HC group (60 people); (2) a LTBI group (60 people); (3) and a TB group (60 people). All participants signed the written informed consent form according to the Declaration of Helsinki, and the protocol was approved by the Ethics Committee of the Beijing Chest Hospital, Capital Medical University. All of them were HIV-negative adults (≥18 years old) and had no TB history before (Supplementary Table S1). We identified TB patients based on positive *Mtb* culture and smear test results. The LTBI patients were identified based on positive tuberculin skin test (TST) and interferon-gamma release assay (IGRA) results, but their other indicators were the same as healthy controls. For HC individuals, they were identified by normal computed tomography (CT) chest films, negative TST and IGRA results. For each group, the serum samples were pooled (*n* = 60) for subsequent experiments.

### Exosome Isolation and Identification

We isolated the serum exosomes as previously described (Lv et al., 2017). We first performed differential centrifugation (1000 g for 10 min at 4°C, and 16,500 g for 30 min at 4°C) to remove the cell debris, followed by ultrafiltration using 0.22 μm filters. We then performed ultracentrifugation (120,000 g for 2 h) to obtain exosome pellets.

Transmission electron microscopy (TEM) combined with immunogold labeling was employed to visualize exosomes (Melo et al., 2015). The exosome pellets were first suspended and dropped onto 200 mesh formvar carbon-coated nickel grids, followed by incubation with 50 mM glycine. After being blocked with 5% bovine serum albumin (BSA), the samples were incubated with rabbit anti-human antibodies (anti-CD9 (SBI, United States), anti-CD63 (SBI, United States), anti-Hsp70 (SBI, United States) and anti-Calreticulin (Abcam, United States)). The samples were then incubated with the goat anti-rabbit secondary antibody conjugated with protein A-gold particles (10 nm) (Bioss, China), followed by negatively stained with 3% phosphotungstic acid for 10 min. The exosome-containing grids were air-dried and observed using a JEM-1400 TEM (JEOL, Japan). In addition, we analyzed the exosome size distribution using nanoparticle tracking analysis (NTA, Malvern, United Kingdom) according to the manufacturer’s instructions.

### Small RNA Sequencing and Data Analysis

Total RNAs were prepared using RNAiso-Plus (TaKaRa, Dalian, China) according to the manufacturer’s protocol. The RNA concentration was assessed using a Qubit^®^ 2.0 Flurometer (Life Technologies, CA, United States), and the RNA integrity and length were measured using the Agilent Bioanalyzer 2100 system (Agilent Technologies, CA, United States) (Supplementary Figure S1).

According to our pre-experiments, no less than 400 ng total RNA per sample was required as input material to generate the small RNA library by using NEBNext^®^ Multiplex Small RNA Library Prep Set for Illumina^®^, (NEB, United States.) following manufacturer’s instructions. Briefly, a NEB 3′ SR Adaptor was directly, and specifically ligated to 3′ end of miRNA, siRNA and piRNA. Then the SR RT Primer hybridized to the excess of 3′ SR Adaptor that remained free after the 3′ ligation reaction, and the single-stranded DNA adaptor was transformed into a double-stranded DNA molecule. A 5′ ends adapter was ligated to 5′ends of miRNAs. Then we used M-MuLV Reverse Transcriptase to synthesize the first strand cDNA followed with PCR amplification using LongAmp Taq 2X Master Mix, SR Primer for illumina and index primer. Finally, PCR products were purified on a polyacrylamide gel, and DNA fragments ranging from 140 to 160 bp were recovered and dissolved in 8 μL elution buffer. Finally, we assessed the library quality on the Agilent Bioanalyzer 2100 system.

The RNA libraries were sequenced on Illumina Hiseq 2500 sequencer and 50 bp single-end reads were generated. We then removed the adaptors, filtered the low quality reads from the raw sequencing data, and obtained clean small RNA reads for subsequent analysis. These clean reads were mapped to human reference genome (hg38) by Bowtie (Langmead et al., 2009). We first identified known miRNAs based on miRBase20.0 (miRBase^1^). Then we used miREvo (Wen et al., 2012) and miRDeep2 (Friedlander et al., 2011) to predict new miRNAs. On the other hand, we identified the small RNAs derived from repetitive genomic region using RepeatMasker database (Tarailo-Graovac and Chen, 2009). Expressions were quantified by transcript per million (TPM) (Zhou et al., 2010). Differential expressions were determined using DEGseq (2010) R package (Wang et al., 2010). The differentially expressed miRNAs were selected as follows: |Fold change|≥ 2 and *q*-value <0.05 (Storey, 2003). The functional analysis of miRNA-targeting mRNAs was performed by using ingenuity pathway analysis (IPA) platform (QIAGEN Inc.,^2^).

## Results

### Differential miRNA Expression Profiles Among the Serum Exosomes From HC, LTBI, and TB Groups

**FIGURE 1:**
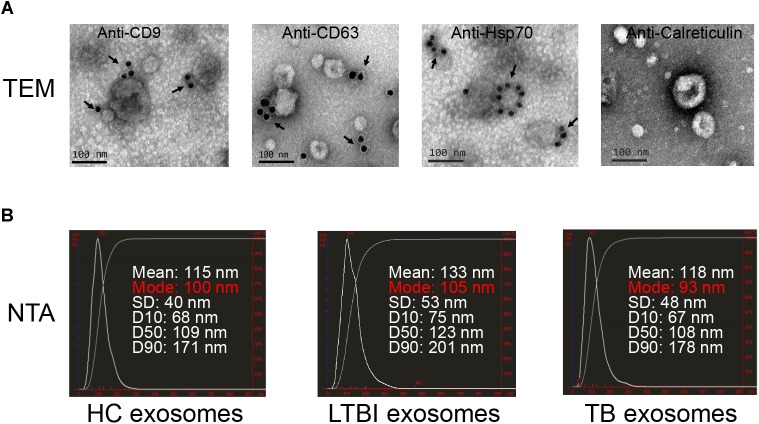
Exosomes quality analysis. **(A)** The images of immunogold electron microscopy showing the marker proteins in exosomes (scale bar = 100 nm). The first three images identified the exosomal marker proteins of CD9, CD63, and Hsp70 by immunogold particles; the last image did not show the expression of calreticulin (an endoplasmic reticulum marker protein) in vesicles. The arrows indicate the golden particles (10 nm). **(B)** The images show the exosome size distribution of HC, LTBI, and TB groups through “nanoparticle tracking analysis” (NTA). The distribution peaks of exosomes are at ∼100 nm.

To explore the expression profiles of miRNAs in serum exosomes derived from HC, LTBI, and TB individuals, we isolated the exosomes as previously described (Lv et al., 2017). The exosomes were then validated by NTA and immunogold electron microscopy analyses. The results indicated that CD9, CD63, and Hsp70 could be detected by the immunogold particles (Figure 1A). As for the negative control, we could not detect calreticulin, an endoplasmic reticulum marker protein in the exosome samples (Figure 1A), which validated the quality of exosomes from the other side. In addition, the NTA image indicates the diameter distribution peaks at ∼100 nm (Figure 1B).

**FIGURE 2:**
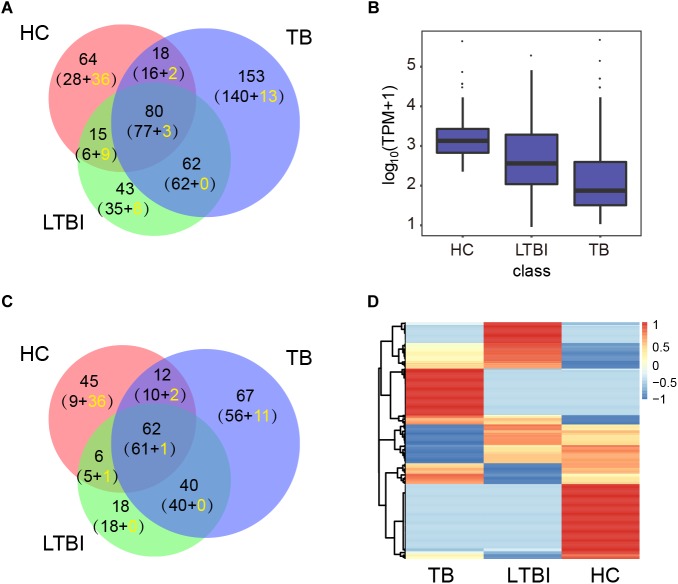
Expression profiling analysis of the miRNA in serum exosomes derived from HC, LTBI, and TB groups. **(A)** Venn diagram showing the overlap of the miRNAs in the three groups. The number in brackets indicates the known and novel (yellow) miRNAs, respectively. **(B)** Box plot showing the expression levels (TPM values) of miRNA in the three groups. **(C)** Venn diagram showing the overlap of the differentially expressed miRNAs among the three groups (|Fold change| ≥ 2, *p* < 0.05, *q* < 0.05). The number in brackets indicates the known and novel (yellow) miRNAs, respectively. **(D)** Heatmap showing the expressions of the differentially expressed miRNAs from three groups (|Fold change|≥ 2, *p* < 0.05, *q* < 0.05). The color key indicates the expression level of the miRNAs.

We then performed high-throughput sequencing of small-RNA transcriptomes (≤50 nt) for miRNA expression profiles. The results identified 177, 200, and 313 miRNAs in HC, LTBI, and TB groups base on miRDeep2 software, including 364 known miRNAs and 71 unreported miRNAs (Figure 2A and Supplementary Table S2). The expression level of miRNAs from high to low is as below: HC, LTBI, and TB groups (Figure 2B). We further obtained the differential expression profiles among the three groups by pairwise comparison (|Fold change|≥ 2, *p* < 0.05 and *q* < 0.05), and identified 250 differentially expressed miRNAs (Figure 2C,D).

### Specifically Expressed miRNAs in Different Groups

**FIGURE 3:**
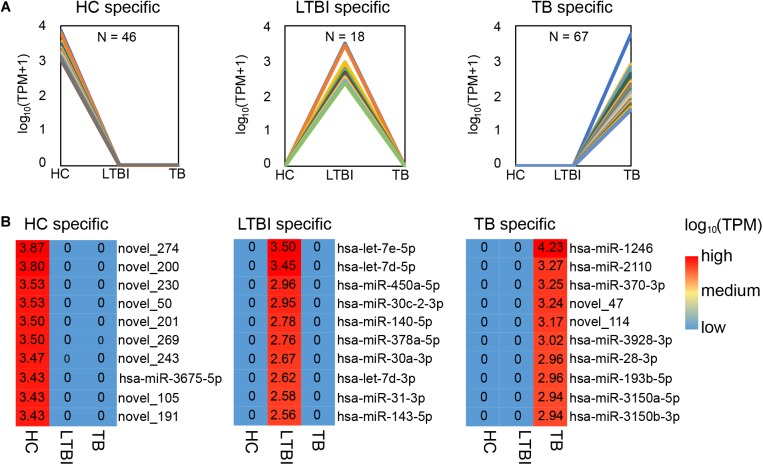
Specifically expressed miRNAs in serum exosomes derived from HC, LTBI and TB groups. **(A)** Specifically expressed miRNAs in the HC, LTBI, and TB groups. **(B)** Heatmap showing the top-10 specifically expressed miRNAs in the HC, LTBI, and TB groups. The color key represents the expression of the top-10 specifically expressed miRNAs range from low (blue) to high (red). The value of log10 (TPM) are shown in the cells.

Among the 250 differentially expressed miRNAs, we identified 45, 18, and 67 specifically expressed miRNAs in HC, LTBI, and TB groups (Figure 3A). We further described the top-10 specifically expressed miRNA in each group (Figure 3B), including 1, 10, and 8 known miRNAs in HC, LTBI, and TB groups.

**FIGURE 4:**
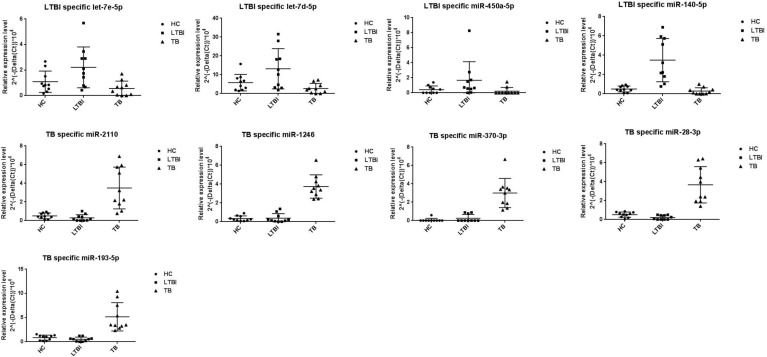
Validation of miRNA candidates from HC, LTBI, and TB exosomal samples. We selected 20 miRNAs among top10 LTBI and TB specific miRNA candidates for qRT-PCR validation. We enrolled ten individuals for HC, LTBI, and TB groups, respectively. We validated 4 and 5 miRNA candidates as potential biomarkers for LTBI and TB.

We further performed the qRT-PCR verification for top-10 specifically expressed miRNAs in 30 samples (10 HC, 10 LTBI, and 10 TB samples) (Figure 4). Four miRNAs were identified to be specifically expressed in LTBI samples (hsa-let-7e-5p, hsa-let-7d-5p, hsa-miR-450a-5p, and hsa-miR-140-5p); five miRNAs were observed to be specifically expressed in TB samples (hsa-miR-1246, hsa-miR-2110, hsa-miR-370-3p, hsa-miR-28-3p, and hsa-miR-193b-5p).

### Differential Expression Panels of the Serum Exosomal miRNAs From LTBI and TB Groups

**FIGURE 5:**
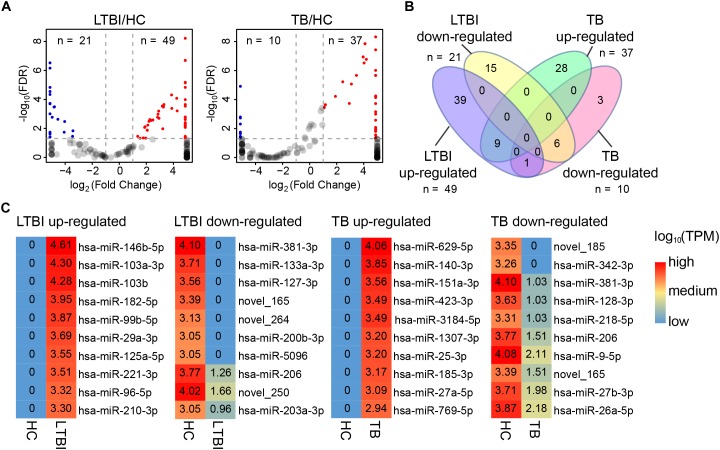
Differential expression panel of exosome miRNA in the LTBI and TB compared with HC groups. **(A)** Volcano plot showing the differentially expressed miRNAs in the LTBI and TB compared with HC. The red dots represent the up-regulated miRNAs, while the blue dots represent the down-regulated miRNAs (|Fold change| ≥ 2, *p* < 0.05, *q* < 0.05). **(B)** Venn diagram showing the different expression panels between the TB and LTBI groups compared with HC (|Fold change|≥ 2, *p* < 0.05, *q* < 0.05). **(C)** Heatmap showing the top-10 differentially expressed miRNAs in LTBI and ATB compared with HC. The color key represents the expression of the top-10 differentially expressed miRNAs range from low (blue) to high (red) expressions. The value of log10 (TPM) are shown in the cells.

In addition to specifically expressed miRNAs, we identified 101 differentially expressed miRNAs of TB and LTBI in comparison with HC groups: 49 up-regulated and 21 down-regulated miRNAs were detected in LTBI; 37 up-regulated and 10 down-regulated miRNAs were identified in TB (Figure 5A,B). Venn diagram exhibited relatively distinct miRNA expression panels between the LTBI and TB groups: 39 and 28 miRNAs were only up-regulated in LTBI and TB, respectively; 15 and 3 miRNAs were uniquely down-regulated in LTBI and TB, respectively; one miRNA (hsa-miR-26a-5p) was up-regulated in LTBI but down-regulated in TB. On the other hand, 9 up-regulated and 6 down-regulated miRNAs were shared in both LTBI and TB groups (Figure 3B).

We further identified top-10 up-/down-regulated miRNAs in LTBI and TB comparing with HC individuals (Figure 5C).

### Six Expression Patterns of Serum Exosomal miRNAs Among the Three Groups

**FIGURE 6:**
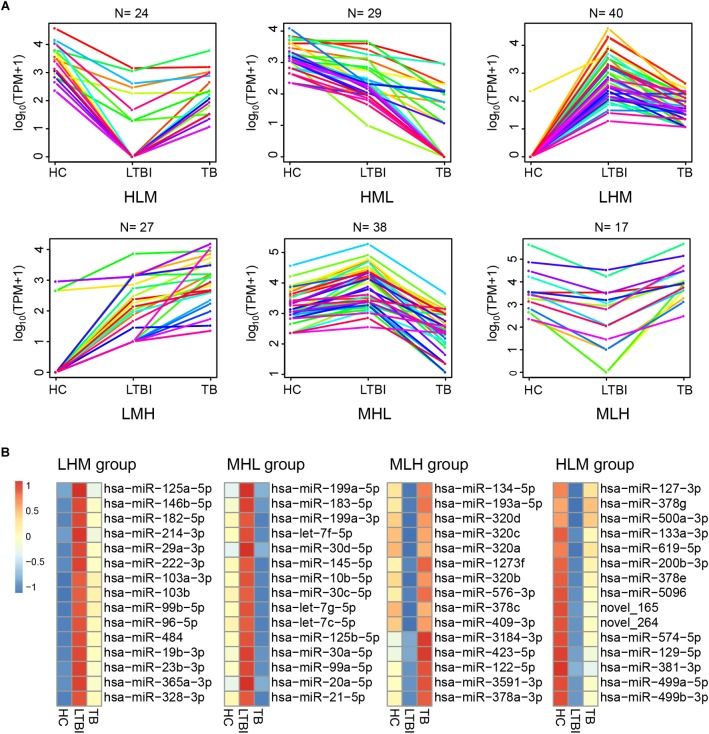
Expression patterns of the differentially expressed miRNAs among the HC, LTBI, and TB groups. **(A)** Six patterns were determined based on TPM values, including LMH, LHM, MHL, HML, MLH, and HLM. (L, low expression level; M, medium expression level; H, high expression level; N, the gene numbers of each pattern). **(B)** Heatmap showing the top-15 high and low differentially expressed miRNAs (LHM, MHL, MLH, and HLM patterns) in the LTBI groups. The color key represents the value of log10 (TPM) from low (blue) to high (red) expressions.

Besides the specifically expressed miRNAs, we classified the differentially expressed miRNAs into six expression patterns based on the miRNA expression trends of the three groups, including MLH, MHL, LMH, LHM, HML, and HLM groups (L, low expression level; M, medium expression level; H, high expression level) (Figure 6A). We first focused on the miRNAs in LMH and HML groups with significance: three miRNAs (hsa-miR-140-3p, hsa-miR-423-3p, and hsa-miR-3184-5p) displayed a successively increased expression trend in HC, LTBI and TB groups with significance (|Fold change|≥ 2, *p* < 0.05 and *q* < 0.05), which might provide potential biomarkers for differentiation of the three groups.

We then screened the top-15 highly or low expressed miRNAs in LHM, MHL, MLH, and HLM groups (Figure 6B), which may provide potential targets of detection/diagnosis for LTBI individuals.

### Small RNAs Derived From Repetitive Region of Genome Account for the Largest Share Among All Kinds of Small RNAs in Exosome

**FIGURE 7:**
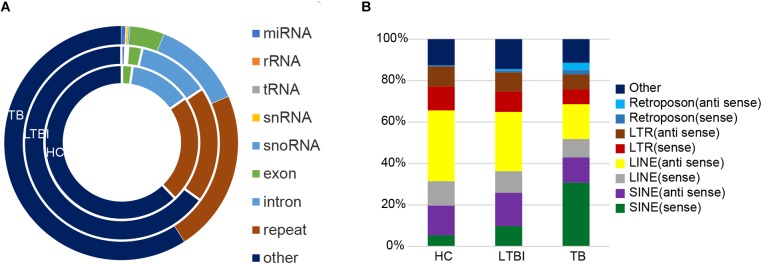
Classification and expression of the small RNAs (≤50 nt) derived from the three groups. **(A)** Classification of small RNAs (≤50 nt) from the three groups. The circles from inner to outer represent various types of small RNAs (shown as different colors) from the three groups. The small RNAs derived from repetitive genomic region account for the largest share (brown) among all kinds of small RNAs (exclude “other” small RNAs). **(B)** Expression level of the small RNAs from various repeated sequences among the three groups.

Except for miRNAs, we also identified many other types of small RNAs in the three groups. Among them, the small RNAs derived from repetitive region of genome account for the largest share among all kinds of small RNAs in exosome (Cha et al., 2015), which include 22.6, 19.4, and 22.4% of total uniquely mapped reads in HC, LTBI, and TB groups, respectively (Figure 7A). In particular, the percentages of small RNAs from sense SINE elements in exosomes gradually increased with *Mtb* infection progresses, (Figure 7B), while the percentages of small RNAs derived from anti-sense LINE elements gradually declined with *Mtb* infection progresses. SINE and LINE RNAs were reported to participate in immune responses to virus infections (Liu et al., 1995; Karijolich et al., 2017; Percharde et al., 2018).

## Discussion

In this study, we revealed differential miRNA expression profiles of serum exosomes derived from HC, LTBI, and TB groups. First, we identified some specifically expressed miRNAs in the three groups among the differentially expressed miRNAs. These provide informative biomarkers for detection of latent and active TB. Furthermore, four and five exosomal miRNAs were validated to be specifically expressed in LTBI (hsa-let-7e-5p, hsa-let-7d-5p, hsa-miR-450a-5p, and hsa-miR-140-5p) and TB samples (hsa-miR-1246, hsa-miR-2110, hsa-miR-370-3P, hsa-miR-28-3p, and hsa-miR-193b-5p), respectively. Additionally, in LTBI groups, we found three specifically expressed miRNAs belonging to the human miRNA lethal 7 (hsa-let-7) family: hsa-let-7d-3p, hsa-let-7d-5p, and hsa-let-7e-5p. Some members of the hsa-let-7 family were reported to play roles in immune response to *Mtb* infection (Fu et al., 2011; Sharbati et al., 2011). In TB groups, several specifically expressed miRNAs (such as hsa-miR-142-3p, hsa-miR-144-3p, and hsa-miR-23a-5p) have also been reported to be closely related to the immune response to *Mtb* infection: hsa-miR-142-3p could modulate phagocytosis by interacting with N-Wasp in host cells (Bettencourt et al., 2013); miR-144-3p could inhibit autophagy activation and enhance the BCG infection by targeting ATG4a in RAW264.7 macrophage cells (Guo et al., 2017); miR-23a-5p could influence autophagy and mycobacterial survival by targeting TLR2 in TLR2/MyD88/NF-κB pathway during *Mtb* infection process (Gu et al., 2017).

Four distinct expression panels were observed among the three groups. The top-10 up-/down-regulated expressed miRNAs in the panels (Figure 5C) included 35 known miRNAs. Among them, three miRNA families have been demonstrated to be associated with *Mtb* infection: hsa-miR-27b-3p, hsa-miR-29a-3p and has-miR-26a-5p (Sharbati et al., 2011; Ahluwalia et al., 2017; Sahu et al., 2017). Specially, miR-27a/b was predicted to target to 3′-UTR of interferon regulatory factor 4 (IRF4) in macrophage, which could lead to the macrophage polarity and conversion to foam cells and further influence survival of *Mtb* (Ahluwalia et al., 2017). The has-miR-29a was reported to affect the apoptosis of *Mtb*-infected macrophages by targeting caspase 7 (Sharbati et al., 2011). The hsa-miR-26a-5p has been documented to regulate innate immune signaling, polarization of macrophages and trafficking of *Mtb* to lysosomes by targeting Krüppel-like transcription factor 4 (KLF4) (Sahu et al., 2017). These differential expression exosomal miRNAs also provide potential detection/diagnose biomarker panels to differentiate LTBI and TB from HC.

Six expression patterns were identified among the three groups. Among the top-15 high and low differentially expressed miRNAs (LHM, MHL, MLH, and HLM patterns) in the LTBI groups (Figure 6B), six miRNAs have also been previously reported to be associated with TB infection, including has-miR-423-5p, has-miR-22-3p, has-miR-483-5p, has-miR-191-5p, has-miR-576-3p, and has-miR-27b-3p (Elkington et al., 2005; Qi et al., 2012; Zhang et al., 2013; Maimon et al., 2014; Meng et al., 2014; Cui et al., 2017).

In addition, our research also revealed some exosomal miRNAs belonging to the same families previously reported in exosome from TB patients, such as miR-93-5p (Fu et al., 2011), miR-29a-3p (Fu et al., 2011), miR-378d (Zhang et al., 2013), miR-378f (Zhang et al., 2013), miR-378i (Zhang et al., 2013), miR-22-3p (Zhang et al., 2013), miR-196b (Zhang et al., 2014) and miR-155-5p (Furci et al., 2013; Zhang et al., 2015; Zhou et al., 2016; Supplementary Table S3).

These specifically expressed exosomal miRNAs and up-/down-regulated exosomal miRNAs in the panels/patterns provided potential biomarkers for the detection of latent and active tuberculosis.

Interestingly, the small RNAs derived from repetitive region of genome account for the largest share among all kinds of small RNAs we identified in exosome. Notably, the percentages of small RNAs from sense SINE elements in exosomes gradually increased with *Mtb* infection progresses (Figure 7B), which is in agreement with previous studies concerning virus infection (Liu et al., 1995; Karijolich et al., 2017). Previous research indicated that expression of SINE elements could be activated or up-regulated under cellular stress such as virus infection (Karijolich et al., 2017). On the other hand, we observed that the percentage of small RNAs derived from sense/anti-sense LINE elements gradually declined with *Mtb* infection progresses.

We also performed functional analyses of exosomal miRNA-targeting mRNAs using IPA software. About 400 functional items were identified in LTBI or TB groups comparing with HC individuals, which belonged to three categories (“Molecular and Cellular Functions,” “Physiological System Development and Function,” and “Disease and Disorders”). We then screened the top-10 items with the biggest change in LTBI and TB groups (Supplementary Figure S2). All of the 10 items displayed a more deteriorated trend in TB than that in LTBI, suggesting a gradual decline in health status with latent TB developing to the active TB.

## Conclusion

In conclusion, our findings provide important reference and improved understanding about miRNAs and repetitive region-derived small RNAs in exosome during *Mtb* infectious process, and facilitate the development of potential molecular targets for detection/diagnosis of latent and active tuberculosis.

## Data Availability

Small RNA sequencing raw data have been deposited at Gene Expression Omnibus (GSE124120).

## Ethics Statement

This study was carried out in accordance with the recommendations of the Helsinki Declaration and its later amendments or comparable ethical standards, the Ethics Committee of the Beijing Chest Hospital, Capital Medical University. This article does not contain any studies with animals performed by any of the authors. The protocol was approved by the Ethics Committee of the Beijing Chest Hospital, Capital Medical University.

## Author Contributions

LL, XZ, FC, and ZZ designed the study. LL, XZ, JW, and LP performed the experiments. LL, XZ, CL, TY, HJ, ZL, QS, and LY performed the bioinformatics analyses. LL, XZ, CL, TY, and FC prepared the manuscript. All authors contributed to and approved the final manuscript.

## Conflict of Interest Statement

The authors declare that the research was conducted in the absence of any commercial or financial relationships that could be construed as a potential conflict of interest.

## Footnotes

1 http://www.mirbase.org/

2 https://www.qiagenbioinformatics.com/products/ingenuity-pathway-analysis
