# Supplementary material for: Small RNA Profiles of Serum Exosomes Derived From Individuals With Latent and Active Tuberculosis
Source: Front Microbiol. 2019 May 28;10:1174. doi: 10.3389/fmicb.2019.01174 (PMC6546874; doi:10.3389/fmicb.2019.01174)
Supplement: Supplementary file 1 [file Table_1.DOCX]

**Supplementary Table S1 The demographic information of the three human subject groups.**

|  | **HC** | **LTBI** | **TB** |
| --- | --- | --- | --- |
| Number | 60 | 60 | 60 |
| Male/Female | 24/36 | 27/33 | 37/23 |
| Mean age ± SD(y) | 38.0±9.1 | 40.2±7.9 | 42.1±8.2 |
| Age range(y) | 20-55 | 23-56 | 19-65 |
| Smokers/Nonsmokers | 15/45 | 18/42 | 19/41 |
