# Supplementary material for: Small RNA Profiles of Serum Exosomes Derived From Individuals With Latent and Active Tuberculosis
Source: Front Microbiol. 2019 May 28;10:1174. doi: 10.3389/fmicb.2019.01174 (PMC6546874; doi:10.3389/fmicb.2019.01174)
Supplement: Supplementary file 4 [file Data_Sheet_1.PDF]

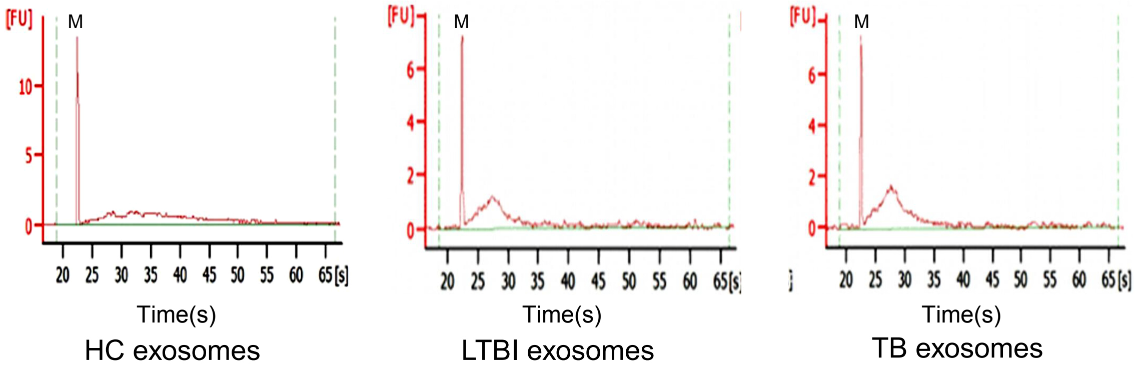

Supplementary Figure S1. The size and integrity analysis of exosomal RNAs by Agilent Bioanalyzer 2100. The diagrams of exosomal RNAs from HC, LTBI and TB groups show the peaks between 25-30s without rRNAs peaks.
