# Supplementary material for: Small RNA Profiles of Serum Exosomes Derived From Individuals With Latent and Active Tuberculosis
Source: Front Microbiol. 2019 May 28;10:1174. doi: 10.3389/fmicb.2019.01174 (PMC6546874; doi:10.3389/fmicb.2019.01174)
Supplement: Supplementary file 5 [file Data_Sheet_2.PDF]

Category	Diseases and Bio Functions	Z-score LTBI/HC	Z-score TB/HC	-log(*p*-value) LTBI/HC	-log(*p*-value) TB/HC
Physiological System Development and Function	Morbidity or mortality	0	8.64	0	7.16
	Organismal death	0	8.53	0	6.97
	Size of body	0	-5.30	0	4.11
	Perinatal death	1.52	5.25	2.41	6.19
Disease and Disorders	Growth failure	1.59	5.20	3.55	5.27
	Aplasia or hypoplasia	1.12	4.53	3.04	6.37
	Hypoplasia	1.12	4.32	2.74	5.14
Molecular and Cellular Functions	Cell movement	-0.92	-4.95	2.59	6.37
	Migration of cells	-1.06	-4.67	2.23	5.46
	Differentiation of embronic stem cells	-2.10	-2.69	2.09	2.70

Z-score scale: High – 0 – Low. -log(*p*-value) scale: High – Low – 0.

Supplementary Figure S2. Functional analysis of miRNA-targeting mRNA using IPA. Heatmap showing the top-10 disease and biological function items (|LTBI Z-score| + |TB Z-score|) of the miRNA-targeting mRNAs (Z-score ≠ 0; Z-score > 0: up-regulation (red); Z-score < 0: down-regulation (blue)). The items covered all the three IPA categories including the "Molecular and Cellular Functions", "Physiological System Development and Function", and "Disease and Disorders". Colors indicate the differential significance level from low (white) to high (orange) (-log (*p*-value) > 1.3: high significance level).
